# Silencing of *RAB3A* Reshapes the Extracellular Vesicle ncRNA Landscape in Diabetic Podocytes and Uncovers miR-126-3p as a Candidate Mediator of Podocyte Injury

**DOI:** 10.3390/biom16101466

**Published:** 2026-10-08

**Authors:** Olga Martinez-Arroyo, Ana Flores-Chova, Lesley Escriva, Sergio Martinez-Hervas, Cristina Grange, Benedetta Bussolati, Maria Jose Forner, Josep Redon, Raquel Cortes, Ana Ortega

**Affiliations:** 1 Cardiometabolic and Renal Risk Research Group, Biomedical Research Institute of Hospital Clinico de Valencia INCLIVA, 46010 Valencia, Spain; aflores@incliva.es (A.F.-C.); lescriva@incliva.es (L.E.); maria.jose.forner@uv.es (M.J.F.); josep.redon@uv.es (J.R.); raquel.cortes@uv.es (R.C.); 2 Service of Endocrinology and Nutrition, Hospital Clinico Universitario of Valencia, 46010 Valencia, Spain; sergio.martinez@uv.es; 3 Cardiometabolic Risk and Diabetes Group, Biomedical Research Institute of Hospital Clinico de Valencia INCLIVA, 46010 Valencia, Spain; 4 Department of Medicine, Faculty of Medicine, University of Valencia, 46010 Valencia, Spain; 5 CIBERDEM (CIBER of Diabetes and Associated Metabolic Diseases), 28029 Madrid, Spain; 6 Department of Medical Sciences, University of Turin, 10126 Turin, Italy; cristina.grange@unito.it (C.G.); benedetta.bussolati@unito.it (B.B.); 7 Internal Medicine Unit, Hospital Clinico Universitario, 46010 Valencia, Spain; 8 CIBEROBN (CIBER of Pathophysiology of Obesity and Nutrition), 28029 Madrid, Spain; 9 CIBERCV (CIBER of Cardiovascular Diseases), 28029 Madrid, Spain

**Keywords:** diabetic nephropathy, podocyte, Rab3A, extracellular vesicle, non-coding RNA, miR-126-3p

## Abstract

Diabetic nephropathy (DN) is a major cause of chronic kidney disease and end-stage renal failure, involving progressive damage to renal cells, including podocytes. Extracellular vesicles (EVs) have emerged as important mediators of intercellular communication in the kidney by transporting bioactive molecules such as non-coding RNAs. Rab3A, a small GTPase involved in vesicle docking and fusion at the plasma membrane, may influence EV biogenesis and the cargo composition. This study investigated the non-coding RNA content of EVs released by podocytes following *RAB3A* silencing under diabetic conditions. Cultured podocytes were transfected with *RAB3A*-specific siRNA and exposed to high glucose concentrations. EVs were isolated from culture media and characterized using complementary techniques, and their non-coding RNA cargo was analyzed via high-throughput sequencing and validated via RT-qPCR. Our results reveal that *RAB3A* silencing under high-glucose conditions induced significant changes in EV-associated non-coding RNAs, including altered levels of miR-126-3p. The context-dependent regulation of miR-126-3p suggests its involvement in IRS1/PI3K-Akt signaling, potentially affecting the balance between protective and pathogenic responses in the diabetic kidney. Overall, these findings provide new insights into the role of Rab3A in shaping EV cargo during diabetic stress and highlight EV-associated non-coding RNAs as potential contributors to the pathophysiology of DN.

## 1. Introduction

Diabetic nephropathy (DN) constitutes one of the principal causes of end-stage renal disease worldwide, and remains a foremost clinical and public-health challenge due to progressive glomerular injury, proteinuria, and the subsequent loss of renal function [1,2]. Despite advances in glycemic control, DN continues to represent a leading cause of morbidity and mortality among diabetic patients, emphasizing the urgent need for novel therapeutic strategies and early biomarkers for detection and intervention [3].

Podocytes are specialized epithelial cells that constitute an essential part of the glomerular filtration barrier. Their structural integrity and survival are essential for glomerular homeostasis. The loss of podocytes or impairment of their specialized functions is a defining feature of DN and contributes directly to proteinuric disease, yet the precise molecular triggers that precipitate podocyte maladaptation in the diabetic milieu remain incompletely resolved [4,5].

Recent work has emphasized the centrality of regulated intracellular trafficking and vesicle-mediated communication in maintaining the slit-diaphragm architecture and podocyte function [6,7]. Within this framework, small GTPases of the Rab family act as master regulators of vesicle docking, trafficking, and fusion. Rab3A, a member of this family, was first documented by Rastaldi et al. within glomerular podocytes. Immunohistochemical and ultrastructural studies demonstrated that Rab3A and its effector Rabphilin-3A are localized around vesicle populations in the foot processes of podocytes in human, rat, and mouse kidneys [8,9]. These early observations support the concept that podocytes possess neuron-like, regulated exocytic machinery.

Our group has previously studied the Rab–Rabphilin system in human podocytes subjected to injurious stimuli and directly interrogated the functional consequences of silencing *RAB3A*. We reported stress-dependent alterations in components of this trafficking axis following glucose overload and angiotensin II exposure [10] and showed that silencing the *RAB3A* system under high-glucose conditions modulates podocyte survival, cytoskeletal organization, and the distribution of CD63-positive vesicles [11]. These findings indicate that Rab-dependent trafficking represents an important determinant of podocyte responses to metabolic stress, but leave open which specific downstream cargoes and signaling pathways are most directly controlled by individual Rab GTPases.

Extracellular vesicles (EVs), small membrane-bound particles secreted by mainly all cell types, carry proteins, lipids, and nucleic acids and constitute a versatile mechanism for intercellular communication [12,13,14]. In the kidney, several cell populations, including tubular epithelial cells, endothelial cells, and podocytes, release EVs that participate in intercellular communication and may serve as sources of urinary and plasma biomarkers of renal injury [15,16]. Small non-coding RNAs (ncRNAs), particularly microRNAs (miRNAs), are enriched in EVs and have been implicated in several physiological and pathophysiological processes, including cardiovascular regulation, immune modulation, and inflammation [17,18,19]. In the context of renal disease, our previous studies, along with those of others, have reported the influence of EV-associated microRNAs on renal cell communication, inflammation, and fibrosis, suggesting a potential role in both the pathogenesis and progression of kidney injury [20,21,22]. Moreover, our group has reported that EV-associated miRNA profiles are altered in conditions such as hypertension and systemic lupus erythematosus [23,24,25].

Given the preceding evidence and recognizing the early demonstration that podocytes harbor Rab3A-associated exocytic machinery, we focused this study specifically on Rab3A. Using cultured human podocytes exposed to hyperglycemic conditions, we silenced *RAB3A* and characterized the small ncRNA composition of secreted EVs, with the aim of determining whether *RAB3A* depletion alters selective cargo loading and thereby modulates intercellular signaling pathways that could contribute to podocyte injury in DN.

## 2. Materials and Methods

### 2.1. Podocyte Cell Maintenance and Culture

Conditionally immortalized human podocytes (AB8/13 cell line), generously provided by Prof. Moin Saleem (Children’s Renal Unit and Academic Renal Unit, University of Bristol, Southmead Hospital, Bristol, UK), were employed in this study. These cells carry a temperature-sensitive SV40 T antigen that promotes proliferation at 33 °C. When the culture temperature is shifted to 37 °C, the antigen becomes inactive, resulting in cell cycle arrest and differentiation [26].

For routine culture, podocytes were seeded in collagen type I-coated flasks or plates (Gibco, Thermo Fisher Scientific, Waltham, MA, USA) and cultured in Roswell Park Memorial Institute 1640 medium (RPMI) supplemented with 10% fetal bovine serum (FBS), 1% penicillin–streptomycin (Biowest, Nuaillé, France), and 1% insulin–transferrin–selenium (ITS; Gibco, Thermo Fisher Scientific, Waltham, MA, USA). Cells were incubated at 33 °C and subcultured when they reached approximately 80% confluence, following trypsin-mediated detachment and reseeding at approximately 70% confluence. To promote differentiation, cells were transferred to 37 °C and cultured for 10–14 days in medium supplemented with 2% FBS. All cultures were maintained in a humidified atmosphere containing 5% CO_2_ and 95% air (BINDER, VWR International, Radnor, PA, USA). Following differentiation, podocytes were used for the subsequent experimental procedures.

### 2.2. RAB3A Gene Silencing and Glucose Treatment

Podocytes were transiently transfected with small interfering RNA (siRNA) directed against the *RAB3A* gene using Lipofectamine™ 3000 (Invitrogen, Thermo Fisher Scientific, Waltham, MA, USA) in serum-free Opti-MEM^®^ medium (Gibco, Thermo Fisher Scientific, Waltham, MA, USA), following the manufacturer’s recommendations [11]. A pre-designed pool of *RAB3A*-specific siRNAs (Silencer™ Select, ID: s11667 y s11668; Ambion, Invitrogen, Thermo Fisher Scientific, Waltham, MA, USA) were utilized for gene silencing. Transfection efficiency was assessed using a Cy3™-labeled negative control siRNA (Invitrogen, Thermo Fisher Scientific, Waltham, MA, USA). Control conditions included cells transfected with a non-targeting (scrambled) siRNA (scr) (Silencer™ Select Negative Control; Invitrogen, Thermo Fisher Scientific, Waltham, MA, USA) and with a GAPDH-targeting siRNA as a positive control (Silencer™ Select GAPDH siRNA, ID: 4404024; Invitrogen, Thermo Fisher Scientific, Waltham, MA, USA). The efficiency of transient *RAB3A* knockdown was confirmed at both the mRNA and protein levels; validation data are available in reference [11] and also provided in the Supplementary Material (Supplementary Figures S1 and S2).

At 24 h post-transfection, the culture medium was exchanged for RPMI containing 1% penicillin–streptomycin and 1% ITS. Cells were then cultured under either normal-glucose (NG; 5.5 mM) or high-glucose (HG; 30 mM) conditions. D-(+)-glucose (Sigma Aldrich, St. Louis, MO, USA) was used to achieve the indicated glucose concentrations.

### 2.3. Transfection of Podocytes with miR-126-3p Mimic

Podocytes were transfected with a synthetic miR-126-3p mimic (hsa-miR-126-3p; Ambion™ mirVana™ miRNA mimic, ID: MC12841 Thermo Fisher Scientific, Waltham, MA, USA) using Lipofectamine™ 3000 (Invitrogen, Thermo Fisher Scientific, Waltham, MA, USA) in Opti-MEM^®^ medium (Gibco, Thermo Fisher Scientific, Waltham, MA, USA), according to the manufacturer’s guidelines. A corresponding negative control miRNA mimic (Ambion™ mirVana™ miRNA mimic Negative Control #1, ID: 4464058; Thermo Fisher Scientific, Waltham, MA, USA) was included in all experiments. Transfections were performed in antibiotic-free medium. After 24 h, the transfection mixture was replaced with fresh culture medium, and glucose treatments were carried out as previously described. Cells were then incubated for an additional 24 h at 37 °C.

### 2.4. EV Isolation via Size Exclusion Chromatography

For EV isolation, 15 mL of conditioned culture medium was processed via size-exclusion chromatography (SEC) using qEVoriginal columns (Izon Science, Lyon, France). Briefly, the collected medium was centrifuged at 2250× *g* for 30 min to remove cellular debris and apoptotic bodies. The clarified supernatant was subsequently concentrated via centrifugation at 4000× *g* for 15 min using Amicon Ultra-15 centrifugal filter units with a 100 kDa molecular weight cut-off (Millipore, Burlington, MA, USA), prior to loading onto the qEVoriginal columns. The concentrated samples were subsequently loaded onto size-exclusion chromatography columns pre-equilibrated with phosphate-buffered saline (PBS). Individual SEC fractions were separately quantified for the particle concentration, and fractions 6–11, which showed the highest particle concentrations, were selected and pooled for subsequent analyses, in agreement with our previous characterization of the EV-enriched eluate and previous reports using SEC [22,27]. These fractions were further concentrated using Amicon Ultra-4 centrifugal filters with a 10 kDa cut-off (Millipore, Burlington, MA, USA) at 4000× *g* for 20 min. Purified EVs were stored at −80 °C until further use.

### 2.5. EV Concentration and Size Assessment

EVs isolated from 15 mL of podocyte supernatants were characterized using the Exoid platform (Izon Science Ltd., Lyon, France), which operates on the principle of tunable resistive pulse sensing (TRPS). Before analyzing the samples, the nanopore was calibrated with standard particles (TKP200, mean diameter 215 nm, Izon Science Ltd., Lyon, France) to establish baseline parameters for particle size and concentration measurements. EV preparations were diluted in 0.22 µm-filtered PBS and introduced into the measurement chamber. The translocation of individual vesicles through the nanopore was controlled by adjusting the pressure, applied voltage, and nanopore stretch to maintain a stable event rate within the optimal detection range. Calibration bead measurements were performed both before and after each run to verify instrument accuracy and reproducibility. All measurements were carried out in triplicate. Data integrity was verified via the continuous monitoring of baseline current stability, background noise, and blockade signal profiles. Particle size and concentration data were collected and processed using Izon Control Suite software (version 3.4; Izon Science Ltd., Lyon, France).

### 2.6. Sample Homogenization, Gel Electrophoresis, and Western Blotting

Podocyte pellets and EVs were lysed using RIPA buffer (Thermo Fisher Scientific, Waltham, MA, USA) supplemented with a protease inhibitor cocktail (Sigma-Aldrich, St. Louis, MO, USA). The lysates were centrifuged at 17,000× *g* for 10 min at 4 °C, and the clarified supernatants were collected. The total protein concentration was quantified using the Lowry assay, employing bovine serum albumin (BSA) as the calibration standard.

Protein extracts were separated via electrophoresis on NuPAGE 4–12% polyacrylamide gels (Invitrogen, Carlsbad, CA, USA) and transferred onto polyvinylidene difluoride (PVDF) membranes. After overnight blocking, membranes were incubated for 2 h at room temperature with the following primary antibodies: rabbit monoclonal anti-phospho-IRS1 Ser 307 (1:1000, Proteintech Europe, STL, Manchester, UK), rabbit monoclonal anti-phospho-Akt Ser 473 (1:500, Cell Signaling Technology, Danvers, MA, USA), mouse monoclonal anti-Akt (1:5000, Proteintech Europe, STL, Manchester, UK), and mouse monoclonal anti- β-actin (1:6000, Sigma Aldrich, St. Louis, MO, USA) was used as a loading control. EV characterization was performed using rabbit monoclonal anti-CD9 (1:1000, Abcam, Cambridge, UK), mouse monoclonal anti-CD63 (1:200, Abcam, Cambridge, UK), mouse monoclonal anti-CD81 (1:250, Abcam, Cambridge, UK), and mouse monoclonal anti-syntenin (1:500, OriGene Technologies, Rockville, MD, USA). Rabbit monoclonal anti-calnexin (1:1000, Abcam, Cambridge, UK) and rabbit monoclonal anti-GM130 (1:2000, Abcam, Cambridge, UK) were included as non-EV cellular markers. Following incubation, membranes were washed with Tris-buffered saline containing 0.1% Tween 20 (TBS-T; 20 mM Tris-HCl and 150 mM NaCl) and subsequently exposed for 1 h at room temperature to alkaline-phosphatase-conjugated secondary antibodies (anti-rabbit IgG or anti-mouse IgG; Sigma-Aldrich, St. Louis, MO, USA). After three washes with TBS-T and a final wash with TBS, immunoreactive bands were visualized using 5-bromo-4-chloro-3-indolyl phosphate/nitro blue tetrazolium (BCIP/NBT; Sigma-Aldrich). The developed membranes were digitized using a high-resolution scanner (HP ScanJet Pro 2600 f1, HP, Palo Alto, CA, USA), and band intensities were quantified using ImageQuant™ 7.0 TL software.

### 2.7. Super-Resolution Microscopy Imaging

To assess the presence of surface tetraspanins on podocyte-derived EVs, super-resolution imaging was performed using a Nanoimager S Mark II microscope (Oxford Nanoimaging (ONI), Oxford, UK) equipped with a 100×, 1.4NA oil immersion objective, an XYZ closed-loop piezo 736 stage, and dual or triple emission channels split at 640 and 555 nm. Sample preparation followed previously established protocols [28,29]. EVs were labeled using antibody reagents from the EV Profiler Kit (ONI, Oxford, UK). Briefly, 7 µL of EV suspension were mixed with 2 µL of blocking buffer PBS containing 5% bovine serum albumin and 1 µL of antibody cocktail comprising anti-CD9-Atto488, anti-CD63-Alexa555, and anti-CD81-Alexa647. The mixture was incubated overnight at 4 °C. The following day, the mixture was deposited onto a pre-coated well and incubated for 1 h to allow vesicle attachment. After, wells were washed twice with PBS and fixed and then supplemented with 10 µL of ONI B-Cubed Imaging Buffer (ONI, Oxford, UK) prior to acquisition. Super-resolution images were captured in direct stochastic optical reconstruction microscopy (dSTORM) mode under total internal reflection fluorescence (TIRF) illumination. Single-molecule localization data were filtered using NimOS software (version 1.18.3; ONI, Oxford, UK) and subsequently processed via the Collaborative Discovery (CODI) online analysis platform (https://alto.codi.bio/, (accessed on 17 December 2024), ONI, Oxford, UK). Drift correction was applied using the version 0.2.3 correction algorithm [29].

### 2.8. RNA Isolation and cDNA Synthesis

RNA was isolated from podocyte cell pellets using the miRNeasy Mini Kit (Qiagen, Hilden, Germany), whereas RNA from podocyte-derived EVs was obtained with the Total Exosome RNA and Protein Isolation kit (Invitrogen, Thermo Fisher Scientific, Waltham, MA, USA). Both large and small RNA species were recovered following the respective manufacturer’s protocols. RNA yield and purity were evaluated using a NanoDrop 2000 spectrophotometer (Thermo Fisher Scientific, Waltham, MA, USA). Extracted RNA was maintained at −80 °C until further analysis.

For mRNA quantification, complementary DNA (cDNA) was synthesized using the Ready-To-Go You-Prime First-Strand Beads kit (GE Healthcare, Buckinghamshire, UK), following the supplier’s protocol. For miRNA expression analyses, 2 µL of total RNA was reverse-transcribed using the TaqMan™ Advanced miRNA cDNA Synthesis Kit (Applied Biosystems, Foster City, CA, USA). All RNA samples were within the manufacturer’s recommended input range of 1–10 ng of total RNA per reaction. The resulting cDNA preparations were stored at −80 °C for subsequent experiments.

### 2.9. Small RNA Library Preparation and Next-Generation Sequencing

Small RNA libraries were generated from 6 μL of total RNA per sample using the Small RNA-Seq Library Prep Kit (Lexogen GmbH, Vienna, Austria), following the manufacturer’s protocol optimized for low-input RNA quantities. After adapter ligation and cDNA amplification, libraries were size-selected to retain fragments between 122 and 200 bp using the Pippin Prep automated DNA size selection system (Sage Science, Beverly, MA, USA). Purified and concentrated libraries were quantified via quantitative real-time PCR (RT-qPCR), and quality was evaluated via capillary electrophoresis on a QIAxcel Advanced System (Qiagen, Hilden, Germany). Individual libraries were normalized to 4 nM, pooled, and sequenced on the HiSeq X™ Ten platform (Illumina, San Diego, CA, USA) using 150-cycle paired-end reads.

Sequencing reads were processed with Trim Galore! for adapter removal and quality trimming (https://www.bioinformatics.babraham.ac.uk/projects/trim_galore/) (accessed on 17 December 2024), followed by alignment to the human reference genome (GRCh38) using STAR [30]. Expression quantification of both small and total RNA species was performed using the featureCounts function from the Rsubread package (Bioconductor version 3.20 (R version 4.4.2) [31]). Transcript annotation relied on the GENCODE database for coding and ncRNAs and on miRBase, piRNAbank, and Ensembl for small RNA classes, including miRNAs, small nuclear RNAs, small nucleolar RNAs, ribosomal RNAs, and piwi RNAs (piRNAs).

Only RNA species with a base mean greater than 5 and detected in at least three samples with one or more reads were retained for downstream analysis. Differential expression analysis was carried out using DESeq2 [32], and *p*-values were adjusted for multiple testing with the Benjamini–Hochberg procedure. False discovery rate (FDR) values below 0.05 were considered statistically significant.

### 2.10. Quantitative Real-Time Polymerase Chain Reaction (RT-qPCR)

Quantitative real-time PCR (RT-qPCR) analyses were performed using a LightCycler^®^ 480 II instrument (Roche, Mannheim, Germany). Primer3 software 2.6.0 was used for the primer design of mRNA targets. Each reaction contained the appropriate primer pair, Qiagen Multiplex PCR Master Mix, LC Green reagent (Qiagen, Hilden, Germany), and 2 µL of diluted cDNA.

For miRNA validation and overexpression studies in podocyte pellets and podocyte-derived EVs, the TaqMan™ Small RNA Assay protocol was employed. In brief, 1.33 µL of diluted cDNA was combined with TaqMan™ Universal Master Mix II (no UNG) and the specific TaqMan™ microRNA assay probe for miR-126-3p (ID: 002228; Applied Biosystems, Foster City, CA, USA). All reactions were carried out in triplicate and included appropriate positive and negative controls, including a non-template control. β-Actin (ACTB) and β2-microglobulin (B2MG) served as endogenous reference genes for mRNA normalization, while cel-miR-39-3p (ID: 000200) was used as an exogenous spike-in control for miRNA assays. Relative expression levels were calculated using the comparative 2^−ΔΔCt^ method and are presented as the fold change (FC) relative to the mean expression of the reference genes.

### 2.11. miRNA Target Predictions

Potential targets of miR-126-3p were predicted using three independent databases with complementary approaches: TargetScanHuman 8.0, miRDB v6.0, and miRTarBase v10.0 (all accessed in March 2025 and restricted to Homo sapiens). For TargetScanHuman, predicted miRNA target genes were obtained based on seed-sequence complementarity and target-site features, including the site type, evolutionary conservation, and contextual targeting efficacy, summarized by the cumulative weighted Context++ score. For miRDB, miRNA targets were predicted using the MirTarget machine-learning algorithm. Predicted interactions were assigned a target score ranging from 0 to 100, and only targets with a confident score >60 were included. For miRTarBase, only targets supported by more than one experimental validation method or reported in more than one publication were retained. A target was considered for further analysis only if it was consistently identified in at least two databases.

### 2.12. Statistical Analysis

Statistical analyses were performed using the edgeR Bioconductor package for sequencing data and GraphPad Prism (v.9.0) and SPSS (v.20) for RT-qPCR and protein analyses. Normality of continuous variables was evaluated with the Shapiro–Wilk test. Data are presented as the mean ± SD of the FC, log_2_ fold change, or normalized arbitrary units for continuous variables and as percentages for categorical variables. Comparisons between two groups for RT-qPCR validation were performed using Student’s *t*-test. For comparisons involving three or more groups, one-way analysis of variance (ANOVA) followed by Tukey’s or Šidák’s multiple-comparisons test was used, as appropriate. Šidák’s test was used for specified pairwise comparisons, whereas Tukey’s test was used for multiple pairwise comparisons between groups. Graphical representations were generated using GraphPad Prism (v.9.0) and the VennDiagram package. *p*-values for sequencing analyses were adjusted using the Benjamini–Hochberg procedure to control for the false discovery rate. A *p*-value < 0.05 was considered statistically significant.

## 3. Results

### 3.1. Podocyte EV Characterization

**Figure 1:**
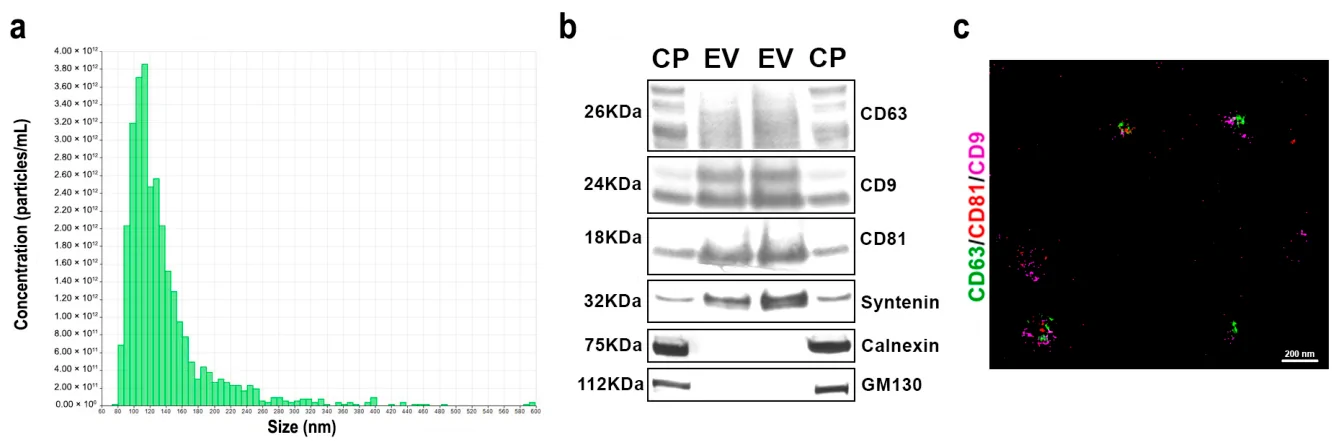
Characterization of podocyte-derived EVs. (**a**) Representative graph from TRPS analysis showing the size distribution and concentration of EVs. (**b**) Images cropped from Western blot membranes showing EV protein markers, the endoplasmic reticulum marker calnexin, and the Golgi apparatus marker GM130. EV fractions expressed the canonical tetraspanins CD63, CD9, and CD81, as well as the late endosomal protein syntenin. (**c**) Super-resolution dSTORM imaging of EVs labeled for CD63 (green), CD81 (red), and CD9 (magenta). The full membranes from panel B are detailed in the Supplementary Material as ESM 1. Scale bar, 200 nm. CP, cell pellet; EV, extracellular vesicle. (Original western blot images see Supplementary Figure S3).

We first characterized the EV preparation to confirm the particle size, protein composition, and membrane marker distribution prior to downstream analyses (Figure 1). Figure 1a shows the TRPS analysis of the isolated EVs. The size distribution is typical of small EVs, with a clear modal population centered at approximately 100–120 nm. Western blot analysis was performed strictly as a qualitative assay to confirm EV identity and purity. We show that EV fractions robustly express the canonical tetraspanins CD63, CD9, and CD81, together with the late endosomal marker syntenin, whereas the endoplasmic-reticulum marker calnexin is detected in the podocyte fraction but is absent in the EV fractions (Figure 1b and Figure S3). Figure 1c shows super-resolution dSTORM imaging of individual vesicles labeled for CD63 (green), CD81 (red), and CD9 (magenta). EVs are observed as discrete nanoscale puncta, with examples of single-label and multi-label puncta and small clusters visible. Together, these data validate the isolation procedure and demonstrate that the material collected corresponds to small EVs displaying canonical tetraspanin markers and endosomal protein content and with a size distribution appropriate for subsequent functional and molecular studies.

### 3.2. Overall Changes in EV Non-Coding RNA Biotypes Following RAB3A Knockdown and High-Glucose Treatment

**Figure 2:**
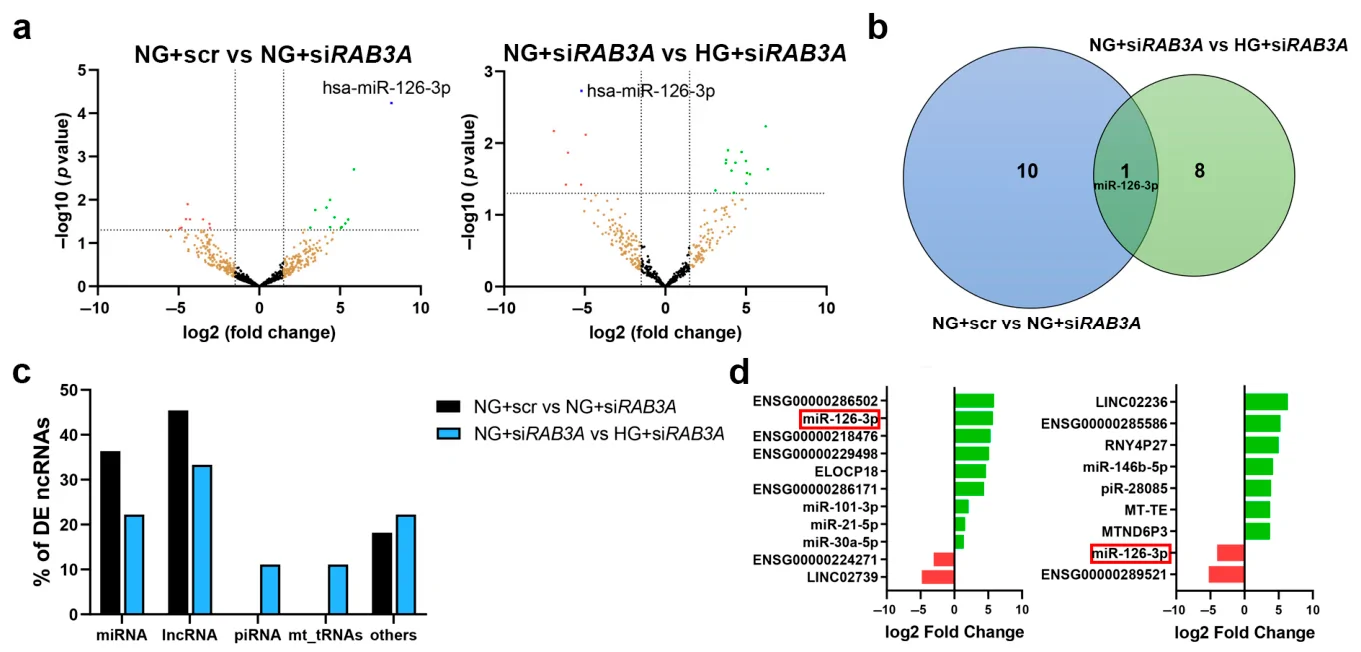
Differential expression of small non-coding RNAs (ncRNAs) in EVs from *RAB3A*-silenced podocytes. (**a**) Volcano plots displaying differentially expressed ncRNAs between NG + scr and NG + si*RAB3A* (left) or NG + si*RAB3A* and HG + si*RAB3A* (right). (**b**) Venn diagram showing the overlap of DE ncRNAs identified in the two comparisons. (**c**) Distribution of DE ncRNA biotypes in NG + scr vs. NG + si*RAB3A* (black) and NG + si*RAB3A* vs. HG + si*RAB3A* (blue). Percentages represent the fraction of all DE ncRNAs assigned to each biotype (miRNA, lncRNA, piRNA, snoRNA, others (processed_pseudogene, miscRNA)). (**d**) Top DE ncRNAs ranked by absolute log2 fold change for NG + scr vs. NG + si*RAB3A* (left) and NG + si*RAB3A* vs. HG + si*RAB3A* (right). Green dots or bars indicate upregulation, and red dots or bars indicate downregulation.

A volcano plot analysis revealed significant alterations in the ncRNA cargo of EVs following *RAB3A* silencing. A comparison between NG + scr and NG + si*RAB3A* identified several ncRNAs with differential expression, including the overexpression of hsa-miR-126-3p (Figure 2a). In contrast, the comparison between NG + si*RAB3A* and HG + si*RAB3A* also highlighted hsa-miR-126-3p, which was downregulated, and additional ncRNAs as significantly dysregulated (Figure 2a). The Venn diagram analysis showed unique overlap between the two experimental conditions, with only hsa-miR-126-3p being commonly altered in both comparisons (Figure 2b). The classification of differentially expressed (DE) ncRNAs by biotype indicated that miRNAs and long ncRNAs (lncRNAs) were the most abundant categories, followed by piRNAs and mitochondrial transfer RNAs, with a smaller proportion assigned to pseudogenes and other classes (Figure 2c). Ranking of the top ncRNAs by fold change showed distinct profiles for each condition (Supplementary Tables S1 and S2). In NG + scr vs. NG + si*RAB3A*, several lncRNAs were downregulated, whereas other biotypes, including miRNAs were upregulated (Figure 2d, left). In NG + si*RAB3A* vs. HG + si*RAB3A*, both upregulated and downregulated ncRNAs were identified, including miRNAs, piRNAs, and lncRNAs (Figure 2d, right). Notably, miR-126-3p was identified as the only ncRNA differentially expressed in both comparisons (NG + scr vs. NG + si*RAB3A* and NG + si*RAB3A* vs. HG + si*RAB3A*), suggesting a common response to *RAB3A* silencing and glucose conditions.

### 3.3. Evaluation of miR-126-3p Expression in Podocytes

**Figure 3:**
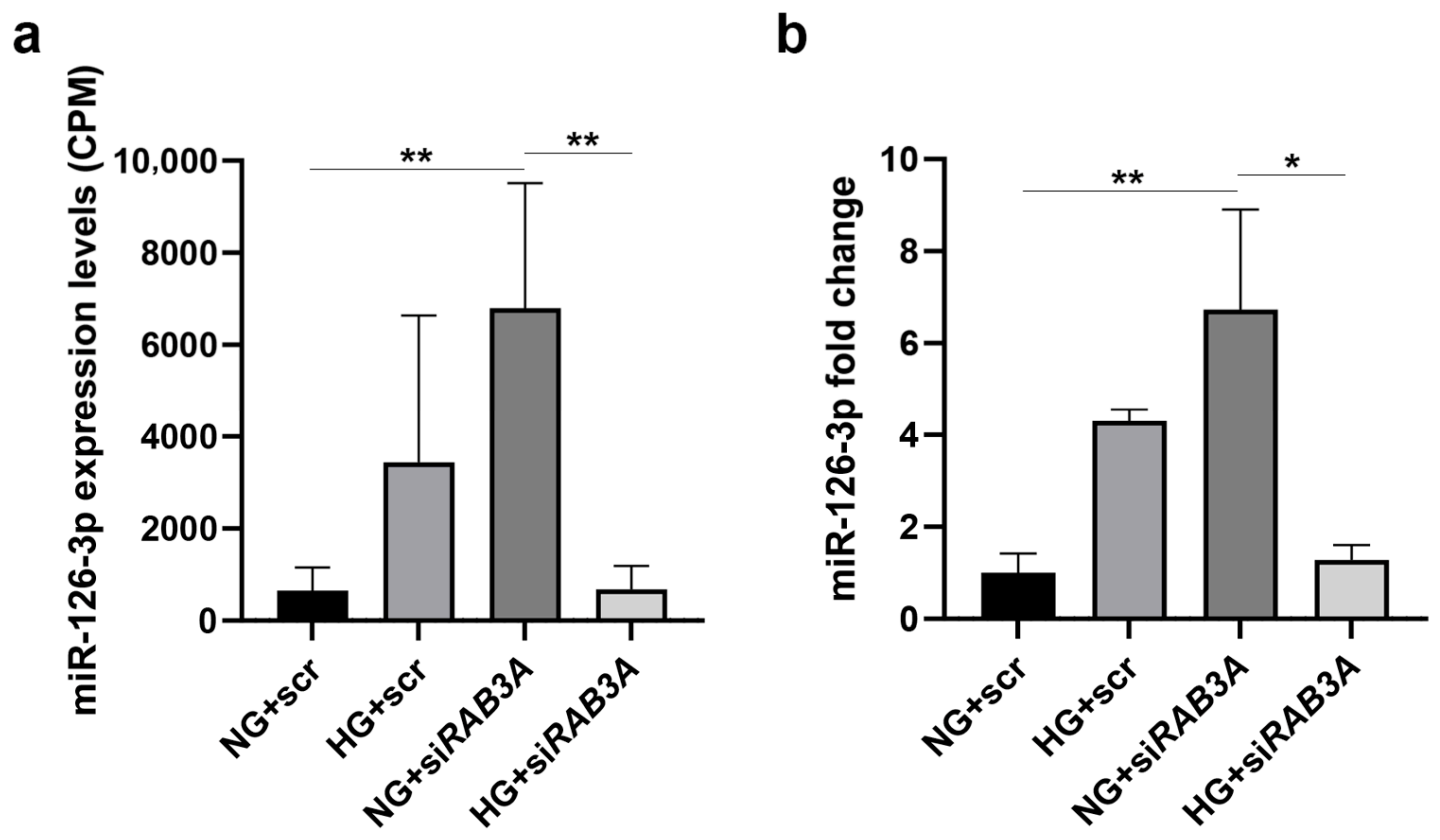
Evaluation of miR-126-3p expression in cultured podocytes. (**a**) Small-RNA-sequencing analysis of miR-126-3p in EVs derived from podocytes silenced and treated with glucose. (**b**) RT-qPCR validation of RNA-sequencing results. Results from RNA sequencing are expressed as CPM (counts per million) and from RT-qPCR are expressed as fold change relative to NG + scr. Sequencing data are presented as the mean ± standard deviation (SD) of the CPM. Differential expression analysis was performed using the DESeq2 package. *p*-values were adjusted for multiple testing using the Benjamini–Hochberg method, and an adjusted *p*-value < 0.05 was considered statistically significant. For RT-qPCR analysis, data are presented as the mean ± SD of the fold change. Statistical significance was assessed using one-way ANOVA followed by Šidák’s multiple comparisons test for selected pairwise comparisons. *p* < 0.05 was considered statistically significant. * *p* < 0.05, ** *p* < 0.01.

RT-qPCR validation of EV miR-126-3p levels supported the sequencing findings: relative to NG + scr, *RAB3A* knockdown in podocytes maintained in NG led to a pronounced increase in EV-associated miR-126-3p (6.73 FC, *p* < 0.05). In contrast, the elevation induced by *RAB3A* silencing was largely diminished when the knockdown cells were cultured under HG conditions (HG + si*RAB3A*), (1.28 FC, *p* < 0.05), (Figure 3).

### 3.4. miR-126-3p Target Identification and Expression and Downstream IRS1/AKT Signaling in Podocytes

Next, we analyzed the miR-126-3p target genes using three independent databases. The complete lists of identified targets are provided in Supplementary TableS S3–S5, and the overlap among the three databases is shown in the Venn diagram (Supplementary Figure S4). Among the common targets, we particularly focused on the insulin receptor substrate-1 gene (*IRS1*), which was consistently identified across all three databases. *IRS1* is involved in insulin resistance, diabetic endothelial dysfunction, and microvascular complications of diabetes, including renal involvement. To further investigate this target, firstly, we conducted gain-of-function assays by transfecting cells with a synthetic miR-126-3p mimic. Successful transfection was verified via the intracellular uptake of Cy3-labeled oligonucleotides, which exhibited strong fluorescence signals (Supplementary Figure S5a). In parallel, RT-qPCR analysis confirmed the marked elevation of miR-126-3p expression in transfected cells compared with negative controls (Supplementary Figure S5b). These findings confirm the efficiency of the transfection procedure and indicate that the subsequent cellular responses could be attributable to miR-126-3p overexpression.

**Figure 4:**
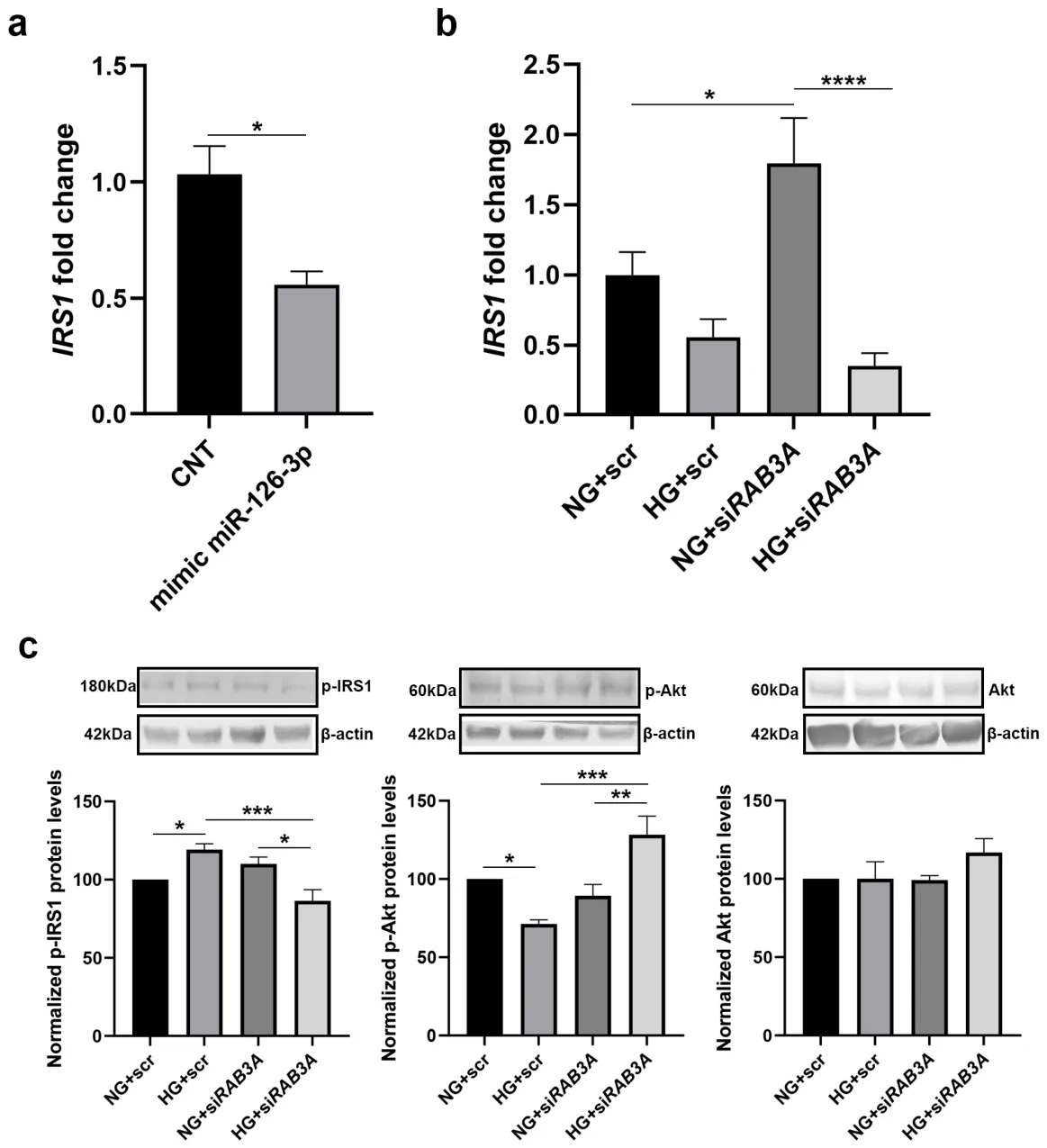
Analysis of the miR-126-3p target IRS1 in stressed and silenced podocytes. (**a**) Evaluation of *IRS1* expression in mimic miR126-3p cultured podocytes. (**b**) IRS1 levels in podocytes transfected with si*RAB3A* and treated with glucose. (**c**) Protein levels of phosphorylated IRS1 (p-IRS1), phosphorylated Akt (p-Akt), and total Akt in podocytes transfected with si*RAB3A* and treated with glucose. mRNA expression levels are presented as the fold change (FC) relative to the NG + scr group. Protein levels were normalized to the NG + scr group, which was set to 100. Statistical significance was assessed using an unpaired Student’s *t*-test for comparisons between two groups or one-way analysis of variance (ANOVA) for comparisons among three or more groups followed by Šidák’s or Tukey multiple comparisons test for pairwise comparisons. *p* < 0.05 was considered statistically significant.* *p* < 0.05; ** *p* < 0.01; *** *p* < 0.001; **** *p* < 0.0001. (Original western blot images see Supplementary Figure S6).

Increasing the levels of miR-126-3p with the mimic led to a marked decrease in *IRS1* mRNA compared with the control of transfection (Figure 4a), indicating that *IRS1* is regulated by miR-126-3p in these cells. In addition, examining *IRS1* expression under different glucose conditions and after *RAB3A* silencing revealed that *IRS1* transcript abundance was affected by both *RAB3A* knockdown and glucose-induced stress (Figure 4b). To further investigate whether these changes were accompanied by alterations in IRS1-related signaling at the protein level, the expression of phosphorylated IRS1 (p-IRS1), phosphorylated Akt (p-Akt), and total Akt was assessed under the different experimental conditions (Figure 4c and Figure S6). p-IRS1 levels were increased in the HG + scr group compared with NG + scr (119.2 ± 8.4; *p* < 0.05). In contrast, *RAB3A* silencing under HG conditions (HG + si*RAB3A*) resulted in a decrease in p-IRS1 levels (86.5 ± 15.8) compared with HG + scr and NG + si*RAB3A* groups (*p* < 0.001 and *p* < 0.05, respectively). Similarly, p-Akt levels showed an opposite pattern, decreasing under HG + scr conditions (71.3 ± 6.1; *p* < 0.05) and increasing following *RAB3A* silencing under HG conditions (HG + si*RAB3A*) (128.3 ± 9.9) compared with HG + scr and NG + si*RAB3A* groups (*p* < 0.001 and *p* < 0.01, respectively). Total Akt protein levels remained unchanged across the different experimental conditions. Together, these results suggest that glucose-induced stress and *RAB3A* silencing modulate the IRS1/AKT signaling pathway in podocytes.

## 4. Discussion

In this study, we demonstrate that silencing *RAB3A* in podocytes exposed to HG conditions causes alterations in EV ncRNA cargo, with miR-126-3p emerging as a common dysregulated miRNA in both group comparisons, untreated and silenced (NG + scr vs. NG + si*RAB3A*) and treated and silenced (NG + si*RAB3A* vs. HG + si*RAB3A*). These findings reveal a previously unrecognized axis linking Rab3A-mediated vesicular trafficking and microRNA-mediated signaling in DN pathogenesis.

Podocyte injury is central to the onset and progression of DN. These terminally differentiated, highly polarized cells rely on tightly regulated vesicular trafficking to preserve slit diaphragm integrity, recycle membrane proteins, and respond to stress [33,34]. Rab GTPases, including Rab3A and Rab27A, are key controllers of vesicle docking and fusion, and their dysregulation may drive podocyte maladaptation in DN. Our previous work showed that HG- or angiotensin II-stressed human podocytes upregulate Rab3A and Rab27A, correlating with podocyte injury and albuminuria in DN patients [10]. Silencing *RAB3A*/*RAB27A* under HG further demonstrated effects on differentiation, cytoskeletal organization, apoptosis, and EV markers like CD63 [11]. These findings underscore a mechanistic role for Rab3A/Rab27A-dependent trafficking as a stress-responsive pathway whose dysregulation contributes to podocyte injury in DN.

EVs are emerging as crucial mediators of intercellular signaling in the kidney. Podocyte-derived EVs have been shown to carry a variety of cargos (proteins, lipids, RNAs), and changes in their release or composition are implicated in disease progression [35]. In our study, we performed a thorough characterization of podocyte-derived EVs, which showed a range of 100–120 nm of size and displayed the classical EV markers typically associated with small EVs. This confirms that the isolated particles represent a well-defined EV population and provides a solid foundation for subsequent functional analyses.

Our transcriptomic analysis shows that depleting *RAB3A* in human podocytes significantly alters the small ncRNA cargo of the secreted EVs, and the identification of miR-126-3p as a principal miRNA altered upon *RAB3A* silencing under HG suggests that Rab3A and glucose stress exerts control over selective miRNA sorting into EVs. This selective loading mechanism also implies that changes in EV-associated ncRNAs may not necessarily mirror proportional changes in their intracellular abundance. Accordingly, although the present study focused on the ncRNA cargo of EVs and did not assess intracellular ncRNA expression in podocytes, future studies should determine whether the altered EV signature correlates with cellular ncRNA levels and elucidate the mechanisms governing Rab3A-dependent ncRNA sorting. miR-126-3p is a well-known regulator of vascular and metabolic pathways, and its dysregulation has been associated with diabetic complications [36,37,38]. In addition, clinical studies show that plasma circulating miR-126 levels decrease in DN patients, and such reductions correlate with albuminuria and the glomerular filtration rate [39]. Interestingly, increased miR-126 levels have been reported in urinary extracellular vesicles from patients with DN [40,41]. These findings suggest that miR-126 is dynamically and compartment-specifically regulated during disease progression, likely reflecting selective loading into extracellular vesicles rather than changes in total circulating miRNA abundance. Experimental evidence indicates that miR-126-3p plays a key role in both endothelial functions and fibrotic responses relevant to renal injury. In models of endothelial-to-mesenchymal transition, a process central to fibrosis, miR-126-3p is downregulated, and its overexpression preserves endothelial markers while reducing mesenchymal markers, both in vitro and in fibrotic renal tissue [42]. Conversely, in a mouse model of unilateral ureteral obstruction (UUO), the overexpression of miR-126 promotes renal fibrosis, enhancing collagen deposition, α-SMA expression, and macrophage infiltration through the inhibition of PI3K/IRS1/FAK signaling, whereas the inhibition of miR-126 attenuates these effects [43]. In accordance with this previous study, our results show that miR-126-3p tends to increase under HG conditions and that this rise becomes even more pronounced when *RAB3A* is silenced in the absence of glucose stress, but in contrast, when *RAB3A* is silenced and cells are exposed to HG, miR-126-3p levels drop markedly. This behavior is consistent with our previous findings, in which *RAB3A* silencing combined with glucose treatment enhanced podocyte differentiation markers, reduced apoptosis, improved cytoskeletal organization, and lowered CD63 exosomal marker expression. Together, these observations suggest that *RAB3A* silencing shifts the cellular response to glucose toward a more protective state and that the downregulation of miR-126-3p under these conditions may form part of the same compensatory program that ameliorates damage.

miR-126-3p has been implicated in the regulation of insulin signaling, notably through interactions with the IRS1/PI3K–Akt pathway. Although the direct targeting of *IRS1* by miR-126-3p has been best characterized in non-renal contexts (e.g., in hepatocytes where the overexpression of miR-126 reduces IRS1 levels and impairs insulin signaling) [44], this mechanistic link is highly relevant to kidney disease. Reduced *IRS1* expression in glomerular cells is associated with diabetic kidney disease, and glomerular IRS1 levels correlate with disease severity and functional outcomes [45]. Therefore, the fact that *RAB3A* silencing in podocytes modifies EV-associated miR-126-3p levels suggests a plausible mechanism in which altered Rab3A functions promote dysregulated EV miR-126-3p release influencing the modulation of IRS1/PI3K-Akt signaling in recipient cells (such as glomerular endothelial, mesangial, or tubular cells), which contributes to insulin resistance, cell stress, or fibrosis in DN. In our study, increasing miR-126-3p levels with a mimic reduced *IRS1* mRNA expression, supporting a regulatory relationship between miR-126-3p and *IRS1* in podocytes. Interestingly, however, miR-126-3p and *IRS1* showed a similar direction of change under HG conditions following *RAB3A* silencing, with both being reduced. Thus, although the mimic experiment supports a regulatory effect of miR-126-3p on *IRS1* expression, the parallel decrease in miR-126-3p and *IRS1* under HG plus *RAB3A* silencing suggests that additional mechanisms are involved in the regulation of *IRS1* under these conditions.

At the protein level, our results further indicate that the IRS1/Akt signaling pathway is affected by both HG exposure and *RAB3A* silencing. HG increased the phosphorylation of IRS1 at Ser307 and was accompanied by a reduction in p-Akt, whereas *RAB3A* silencing under HG conditions decreased IRS1 Ser307 phosphorylation and increased p-Akt, without changes in total Akt. Previous studies have shown that HG can impair insulin signaling in podocytes and glomeruli, including alterations in IRS1 phosphorylation and reduced Akt activation [46]. In addition, studies in podocytes have linked increased IRS1 Ser307 phosphorylation with reduced insulin-stimulated Akt activation, supporting a relationship between IRS1 serine phosphorylation and impaired downstream insulin signaling under metabolic stress [47]. The increase in IRS1 Ser307 phosphorylation observed under HG conditions in our study is therefore consistent with previous evidence associating this modification with altered insulin signaling. However, the functional significance of IRS1 Ser307 phosphorylation is context-dependent. Although early studies proposed that phosphorylation at this site interferes with IRS1 interaction with the insulin receptor and inhibits downstream PI3K signaling [48], subsequent in vivo evidence demonstrated that Ser307 can also have a positive regulatory role in maintaining insulin sensitivity [49]. Therefore, the reduction in IRS1 Ser307 phosphorylation together with the increase in p-Akt observed after *RAB3A* silencing should be interpreted as a change in the IRS1/Akt signaling profile rather than as definitive evidence of improved insulin sensitivity. A further assessment of the activation of IRS1 tyrosine phosphorylation would be required to determine the functional consequences of these changes more precisely.

Together, these studies underscore that miR-126-3p is a critical regulator of endothelial and fibrotic processes in the kidney, with context-dependent effects that may be protective or pathogenic depending on the specific cellular and stress conditions. As described above, the net effect of altering EV-miR-126-3p depends critically on the context. miR-126 has been described as both protective (e.g., supporting endothelial integrity) and potentially deleterious (when suppressing IRS1 under conditions of insulin resistance). The directionality of its change in EVs from *RAB3A*-silenced podocytes could reflect a compensatory or maladaptive response: perhaps silencing *RAB3A* reduces the harmful over-secretion of miR-126-3p under hyperglycemia or conversely disrupts a protective signal that maintains insulin signaling in the glomerulus. In this context, the changes observed in IRS1/Akt signaling following *RAB3A* silencing provide additional evidence that RAB3A-dependent processes are associated with the modulation of intracellular metabolic signaling. However, whether these changes are directly related to the altered EV-associated miR-126-3p levels remains to be established. Additionally, the mechanism by which *RAB3A* silencing alters miR-126-3p loading into EVs is not yet clear. It could involve changes in multivesicular body maturation, alterations in the miRNA-sorting machinery, or the differential trafficking of specific miRNA-loaded vesicles. Dissecting these possibilities will be critical for understanding how vesicular trafficking influences miRNA cargo specificity.

Our in vitro model has inherent limitations. First, the use of transient transfection narrows the time window in which silencing effects can be evaluated, thereby limiting the duration of experimental observations. Additionally, although the experiments were conducted using an immortalized human podocyte line that recapitulates key features of DN, this in vitro system cannot fully replicate the complexity of the in vivo environment. Moreover, although previous studies have shown that osmotic controls do not reproduce the molecular alterations induced by high glucose in podocytes, the absence of an osmotic control in our experimental design should be acknowledged as a limitation of the study. Future validation using podocyte-specific *RAB3A* silencing in animal models will be essential to reinforce the translational relevance of these findings. Despite these limitations, this study provides a valuable foundation for further research aimed at elucidating the role of *RAB3A* silencing and its impact on vesicular trafficking in DN. While we show changes in EV miRNA cargo after *RAB3A* silencing, we have not yet demonstrated functional uptake by recipient renal cell types, nor have we confirmed that the delivered miR-126-3p modulates IRS1 expression or downstream signaling in those cells, moreover taking into account that there are other miRNAs targeting *IRS1*.

## 5. Conclusions

In conclusion, our study demonstrates that *RAB3A* silencing in podocytes under HG conditions significantly alters the ncRNA composition of secreted EVs, with miR-126-3p emerging as a key dysregulated miRNA. These findings reveal a novel Rab3A–miRNA axis linking vesicular trafficking to microRNA-mediated signaling in DN. The context-dependent regulation of miR-126-3p suggests a role in modulating IRS1/PI3K–Akt signaling and influencing the balance between protective and pathogenic responses in the diabetic kidney (Graphical abstract). While further in vivo studies are needed to confirm these mechanisms and their impact on recipient renal cells, this work provides a foundation for exploring Rab3A-dependent EV trafficking as a potential therapeutic target and biomarker in DN.

## Data Availability

The raw small RNA-seq data used in this study are available in the GEO repository under accession number GSE310639 (https://www.ncbi.nlm.nih.gov/geo/query/acc.cgi?acc=GSE310639) (accessed on 17 November 2025).

## Supplementary Materials

The following supporting information can be downloaded at: https://www.mdpi.com/article/10.3390/biom16101466/s1, Figure S1: Evaluation of Rab3A protein knockdown in transfected podocytes; Figure S2: Uncropped Western blot membranes corresponding to Figure S1; Figure S3: Full western blot membranes corresponding to Figure 1; Figure S4: Venn diagram representing the common and unique predicted microRNA target genes across three distinct databases; Figure S5: Assessment of miR-126-3p transfection efficiency in cultured podocytes; Figure S6: Full western blot membranes corresponding to Figure 4; Table S1: List of differentially expressed ncRNA in EVs from podocytes comparing NG scr vs NG siRAB3A groups; Table S2: List of differentially expressed ncRNA in EVs from podocytes comparing NG siRAB3A vs HG siRAB3A groups; Table S3: TargetScanHuman prediction of candidate target mRNAs regulated by miR-126-3p; Table S4: Bioinformatic prediction of potential miR-126-3p target genes using the miRDB database; Table S5: Experimentally validated miR-126-3p target genes retrieved from the miRTarBase database categorized by evidence level.

## Abbreviations

The following abbreviations are used in this manuscript:

ACTB	β-Actin
ANOVA	One-way analysis of variance
B2MG	β2-Microglobulin
BSA	Bovine serum albumin
cDNA	Complementary DNA
DE	Differentially expressed
DN	Diabetic nephropathy
EV	Extracellular vesicle
FC	Fold change
FDR	False discovery rate
FBS	Fetal bovine serum
HG	High glucose
IRS1	Insulin receptor substrate-1
ITS	Insulin-transferrin-selenium
lncRNAs	Long non-coding RNAs
miRNAs	MicroRNAs
ncRNAs	Non-coding RNAs
NG	Normal glucose
p-IRS	Phosphorylated IRS
p-Akt	Phosphorilated Akt
PBS	Phosphate buffered saline
piRNAs	Piwi-RNAs
PVDF	Polyvinylidene difluoride
RT-qPCR	Quantitative real-time PCR
RPMI	Roswell Park Memorial Institute 1640 (cell culture medium)
Scr	Scrambled siRNA
SEC	Size exclusion chromatography
SD	Standard deviation
siRNAs	Small interfering RNA
TBS-T	Tris-buffered saline containing Tween 20
TIRF	Total internal reflection fluorescence
TRPS	Tunable resistive pulse sensing
