# Supplementary material for: Silencing of RAB3A Reshapes the Extracellular Vesicle ncRNA Landscape in Diabetic Podocytes and Uncovers miR-126-3p as a Candidate Mediator of Podocyte Injury
Source: Biomolecules. 2026 Oct 8;16(10):1466. doi: 10.3390/biom16101466 (PMC13702373; doi:10.3390/biom16101466)
Supplement: Supplementary file 1 [file biomolecules-16-01466-s001.zip › biomolecules-4431433-supplementary.pdf]

Article

Silencing of *RAB3A* Reshapes the Extracellular Vesicle ncRNA Landscape in Diabetic Podocytes and Uncovers miR-126-3p as a Candidate Mediator of Podocyte Injury

Olga Martinez-Arroyo [1,*,†], Ana Flores-Chova [1,†], Lesley Escriva [1], Sergio Martinez-Hervas [2,3,4,5], Cristina Grange [6], Benedetta Bussolati [6], Maria Jose Forner [1,4,7], Josep Redon [1,8], Raquel Cortes [1,5,‡] and Ana Ortega [1,9,*,‡]

1 Cardiometabolic and Renal Risk Research Group, Biomedical Research Institute of Hospital Clinico de Valencia INCLIVA, 46010 Valencia, Spain; aflores@incliva.es (A.F.-C.); lescriva@incliva.es (L.E.); maria.jose.forner@uv.es (M.J.F.); josep.redon@uv.es (J.R.); raquel.cortes@uv.es (R.C.)
2 Service of Endocrinology and Nutrition, Hospital Clinico Universitario of Valencia, 46010 Valencia, Spain; sergio.martinez@uv.es
3 Cardiometabolic Risk and Diabetes Group, Biomedical Research Institute of Hospital Clinico de Valencia INCLIVA, 46010 Valencia, Spain
4 Department of Medicine, Faculty of Medicine, University of Valencia, 46010 Valencia, Spain
5 CIBERDEM (CIBER of Diabetes and Associated Metabolic Diseases), 28029 Madrid, Spain
6 Department of Medical Sciences, University of Turin, 10126 Turin, Italy; cristina.grange@unito.it (C.G.); benedetta.bussolati@unito.it (B.B.)
7 Internal Medicine Unit, Hospital Clinico Universitario, 46010 Valencia, Spain
8 CIBEROBN (CIBER of Pathophysiology of Obesity and Nutrition), 28029 Madrid, Spain
9 CIBERCV (CIBER of Cardiovascular Diseases), 28029 Madrid, Spain
* Correspondence: omartinez@incliva.es (O.M.-A.); aortega@incliva.es (A.O.)
† These authors contributed equally to this work.
‡ These authors also contributed equally to this work.

SUPPLEMENTARY MATERIAL

Supplementary Table S1. List of differentially expressed ncRNA in EVs from podocytes comparing NG scr vs NG si*RAB3A* groups.

ID	Symbol	ncRNA biotype	Log2 FC	P-value
ENSG00000286502.1	ENSG00000286502	lncRNA	5.858	0.0019
hsa-miR-126-3p	miR-126-3p	miRNA	5.693	0.0005
ENSG00000218476.2	ENSG00000218476	pseudogene	5.327	0.0352
ENSG00000229498.1	ENSG00000229498	lncRNA	5.108	0.0426
ENSG00000237622.1	ELOCP18	pseudogene	4.659	0.0254
ENSG00000286171.1	ENSG00000286171	lncRNA	4.378	0.0101
hsa-miR-101-3p	miR-101-3p	miRNA	2.076	0.0152
hsa-miR-21-5p	miR-21-5p	miRNA	1.589	0.0172
hsa-miR-30a-5p	miR-30a-5p	miRNA	1.395	0.0495
ENSG00000224271.7	ENSG00000224271	lncRNA	-3.055	0.0448
ENSG00000255008.4	LINC02739	lncRNA	-4.821	0.0440

Supplementary Table S2. List of differentially expressed ncRNA in EVs from podocytes comparing NG si*RAB3A* vs HG si*RAB3A* groups

ID	Symbol	ncRNA biotype	Log2 FC	P-value
ENSG00000251365.5	LINC02236	lncRNA	6.347	0.0230
ENSG00000285586.1	ENSG00000285586	lncRNA	5.254	0.0271
ENSG00000200211.1	RNY4P27	Misc-RNA	4.995	0.0177
hsa-miR-146b-5p	miR-146b-5p	miRNA	4.153	0.0451
hsa-piR-28085	piR-28085	piRNA	3.882	0.0126
ENSG00000210194.1	MT-TE	Mt-tRNA	3.770	0.0172
ENSG00000254132.1	MTND6P3	pseudogene	3.748	0.0191
hsa-miR-126-3p	miR-126-3p	miRNA	-4.017	0.0022
ENSG00000289521.1	ENSG00000289521	lncRNA	-5.232	0.0379

Supplementary Table S3. TargetScanHuman prediction of candidate target mRNAs regulated by miR-126-3p.

**Target gene**	**Representative transcript**	**Gene name**	**Representative miRNA**	**Cumulative weighted context++ score**
PLXNB2	ENST00000449103.1	plexin B2	hsa-miR-126-3p	-0.58
RGS3	ENST00000374140.2	regulator of G-protein signaling 3	hsa-miR-126-3p	-0.45
ADAM9	ENST00000487273.2	ADAM metallopeptidase domain 9	hsa-miR-126-3p	-0.44
HERPUD1	ENST00000379792.2	homocysteine-inducible, endoplasmic reticulum stress-inducible, ubiquitin-like domain member 1	hsa-miR-126-3p	-0.43
KANK2	ENST00000586659.1	KN motif and ankyrin repeat domains 2	hsa-miR-126-3p	-0.42
SLC7A5	ENST00000565644.1	solute carrier family 7 (amino acid transporter light chain, L system), member 5	hsa-miR-126-3p	-0.41
FBXO33	ENST00000298097.7	F-box protein 33	hsa-miR-126-3p	-0.41
PLK2	ENST00000274289.3	polo-like kinase 2	hsa-miR-126-3p	-0.41
SPRED1	ENST00000299084.4	sprouty-related, EVH1 domain containing 1	hsa-miR-126-3p	-0.41
EGFL7	ENST00000371699.1	EGF-like-domain, multiple 7	hsa-miR-126-3p	-0.38
CRK	ENST00000398970.5	v-crk avian sarcoma virus CT10 oncogene homolog	hsa-miR-126-3p	-0.36
CAMSAP1	ENST00000389532.4	calmodulin regulated spectrin-associated protein 1	hsa-miR-126-3p	-0.35
IRS1	ENST00000305123.5	insulin receptor substrate 1	hsa-miR-126-3p	-0.35
BAK1	ENST00000442998.2	BCL2-antagonist/killer 1	hsa-miR-126-3p	-0.32
PEX5	ENST00000266563.5	peroxisomal biogenesis factor 5	hsa-miR-126-3p	-0.32
DIP2C	ENST00000280886.6	DIP2 disco-interacting protein 2 homolog C (Drosophila)	hsa-miR-126-3p	-0.3
SDC2	ENST00000302190.4	syndecan 2	hsa-miR-126-3p	-0.29
ITGA6	ENST00000375221.2	integrin, alpha 6	hsa-miR-126-3p	-0.28
EFHD2	ENST00000375980.4	EF-hand domain family, member D2	hsa-miR-126-3p	-0.27
GNA13	ENST00000439174.2	guanine nucleotide binding protein (G protein), alpha 13	hsa-miR-126-3p	-0.26
TRIM46	ENST00000392451.2	tripartite motif containing 46	hsa-miR-126-3p	-0.25
PCDH7	ENST00000543491.1	protocadherin 7	hsa-miR-126-3p	-0.24
GBP2	ENST00000370466.3	guanylate binding protein 2, interferon-inducible	hsa-miR-126-3p	-0.24
AKAP13	ENST00000394518.2	A kinase (PRKA) anchor protein 13	hsa-miR-126-3p	-0.21
TRAF7	ENST00000326181.6	TNF receptor-associated factor 7, E3 ubiquitin protein ligase	hsa-miR-126-3p	-0.2
LRP6	ENST00000261349.4	low density lipoprotein receptor-related protein 6	hsa-miR-126-3p	-0.19
PTPN9	ENST00000306726.2	protein tyrosine phosphatase, non-receptor type 9	hsa-miR-126-3p	-0.09
SAMD14	ENST00000330175.4	sterile alpha motif domain containing 14	hsa-miR-126-3p	-0.01

Target genes were predicted using TargetScanHuman (V8.0). The cumulative weighted context++ score are detailed for each interaction. Higher negative scores indicate a higher probability of effective miRNA-mRNA repression.

Supplementary Table S4. Bioinformatic prediction of potential miR-126-3p target genes using the miRDB database.

Target Rank	Target Score	miRNA Name	Gene Symbol	Gene Description
1	91	hsa-miR-126-3p	*CAMSAP1*	calmodulin regulated spectrin associated protein 1
2	84	hsa-miR-126-3p	*PTPN9*	protein tyrosine phosphatase, non-receptor type 9
3	83	hsa-miR-126-3p	*SLC7A5*	solute carrier family 7 member 5
4	77	hsa-miR-126-3p	*PIK3R2*	phosphoinositide-3-kinase regulatory subunit 2
5	75	hsa-miR-126-3p	*ADAM9*	ADAM metallopeptidase domain 9
6	73	hsa-miR-126-3p	*PMM1*	phosphomannomutase 1
7	71	hsa-miR-126-3p	*IRS1*	insulin receptor substrate 1
8	71	hsa-miR-126-3p	*SPRED1*	sprouty related EVH1 domain containing 1
9	70	hsa-miR-126-3p	*CRK*	CRK proto-oncogene, adaptor protein
10	67	hsa-miR-126-3p	*RGS3*	regulator of G protein signaling 3
11	66	hsa-miR-126-3p	*RNF165*	ring finger protein 165
12	64	hsa-miR-126-3p	*SLC15A4*	solute carrier family 15 member 4
13	64	hsa-miR-126-3p	*KANK2*	KN motif and ankyrin repeat domains 2
14	57	hsa-miR-126-3p	*TSC1*	TSC complex subunit 1
15	55	hsa-miR-126-3p	*KCNAB3*	potassium voltage-gated channel subfamily A regulatory beta subunit 3
16	55	hsa-miR-126-3p	*DIP2C*	disco interacting protein 2 homolog C
17	52	hsa-miR-126-3p	*ITGA6*	integrin subunit alpha 6
18	51	hsa-miR-126-3p	*PLXNB2*	plexin B2
19	50	hsa-miR-126-3p	*LARP6*	La ribonucleoprotein domain family member 6

Target genes were predicted using the miRDB online database. A Target Score threshold of > 50 was applied to ensure the selection of candidate targets with moderate-to-high statistical confidence. Higher scores indicate a greater probability of a true miRNA-mRNA functional interaction.

Supplementary Table S5. Experimentally validated miR-126-3p target genes retrieved from the miRTarBase database categorized by evidence level.

ID	miRNA	Target	Score
MIRT000343	hsa-miR-126-3p	*SPRED1*	A
MIRT000344	hsa-miR-126-3p	*PLK2*	A
MIRT000798	hsa-miR-126-3p	*SLC45A3*	B
MIRT000965	hsa-miR-126-3p	*CCNE2*	C
MIRT000966	hsa-miR-126-3p	*RGS3*	C
MIRT000967	hsa-miR-126-3p	*TOM1*	B
MIRT001918	hsa-miR-126-3p	*HOXA9*	B
MIRT002993	hsa-miR-126-3p	*CRK*	B
MIRT003428	hsa-miR-126-3p	*VEGFA*	A
MIRT003894	hsa-miR-126-3p	*PIK3R2*	A
MIRT004081	hsa-miR-126-3p	*VCAM1*	B
MIRT004355	hsa-miR-126-3p	*IRS1*	A
MIRT007269	hsa-miR-126-3p	*MMP7*	C
MIRT007381	hsa-miR-126-3p	*CXCL12*	C
MIRT021281	hsa-miR-126-3p	*CRKL*	D
MIRT021282	hsa-miR-126-3p	*PGR*	C
MIRT005020	hsa-miR-126-3p	*E2F1*	C
MIRT005370	hsa-miR-126-3p	*SOX2*	B
MIRT005538	hsa-miR-126-3p	*TWF1*	B
MIRT005540	hsa-miR-126-3p	*TWF2*	B
MIRT005727	hsa-miR-126-3p	*PTPN7*	B
MIRT005728	hsa-miR-126-5p	*PTPN7*	B
MIRT005729	hsa-miR-126-3p	*DNMT1*	B
MIRT006450	hsa-miR-126-3p	*KRAS*	B
MIRT006543	hsa-miR-126-3p	*IGFBP2*	B
MIRT006544	hsa-miR-126-3p	*PITPNC1*	B
MIRT006545	hsa-miR-126-3p	*MERTK*	B
MIRT006558	hsa-miR-126-3p	*EGFL7*	A
MIRT006679	hsa-miR-126-3p	*SLC7A5*	A
MIRT006831	hsa-miR-126-3p	*PIK3CG*	B
MIRT006883	hsa-miR-126-3p	*TEK*	B
MIRT007266	hsa-miR-126-3p	*ADAM9*	B

Target genes were classified according to the experimental evidence scores provided by miRTarBase as follows: Score A represents strong low-throughput evidence, including direct functional assays such as reporter assays (luciferase), Western blot, and RT-qPCR. Score B corresponds to high-throughput functional evidence, comprising large-scale expression microarrays and proteomic analyses. Score C denotes high-throughput binding evidence, indicating physical miRNA–mRNA interactions identified via Next-Generation Sequencing (NGS) methodologies (such as CLIP-seq, HITS-CLIP, and PAR-CLIP) and Cross-linking Ligation and Sequencing of Hybrids (CLASH) techniques.

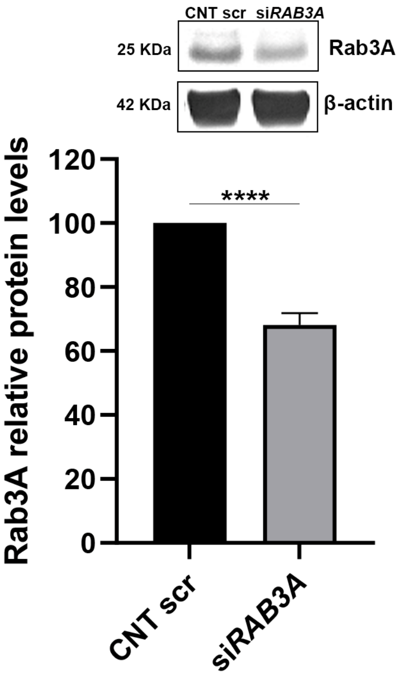

Figure S1. Evaluation of Rab3A protein knockdown in transfected podocytes. Representative Western blot membranes and densitometric quantification showing Rab3A expression levels relative to β-actin (loading control). CNT scr: control scramble cells; si*RAB3A*: *RAB3A*-silenced cells. Data are expressed as mean ± SEM (standard error of the mean).N = 5 independent biological replicates per group. Protein levels were normalized to the control group (100%). Statistical significance is indicated as ****p < 0.0001.

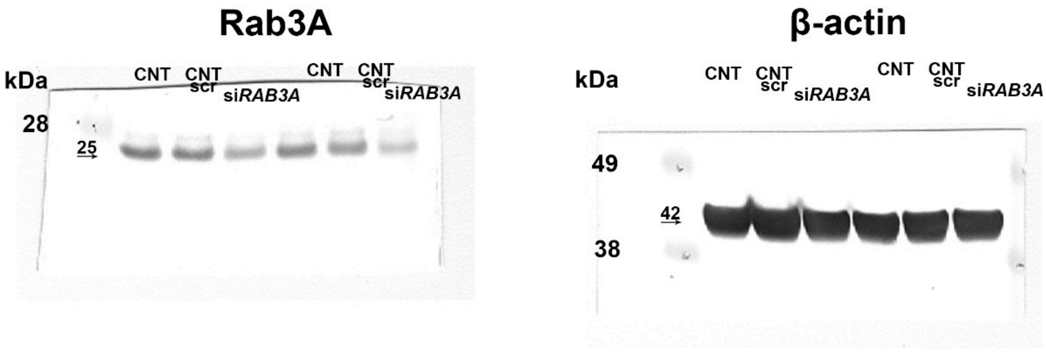

Figure S2. Uncropped Western blot membranes corresponding to Figure S1. Full-length immunoblots showing Rab3A and β-actin protein levels in podocyte cell pellets from control (CNT), control scramble (CNT scr), and R*AB3A*-silenced (si*RAB3A*) groups. Molecular weight markers (expressed in kDa) are indicated on the left of each blot. Arrows, along with the corresponding values above, denote the specific molecular weight of the proteins of interest.

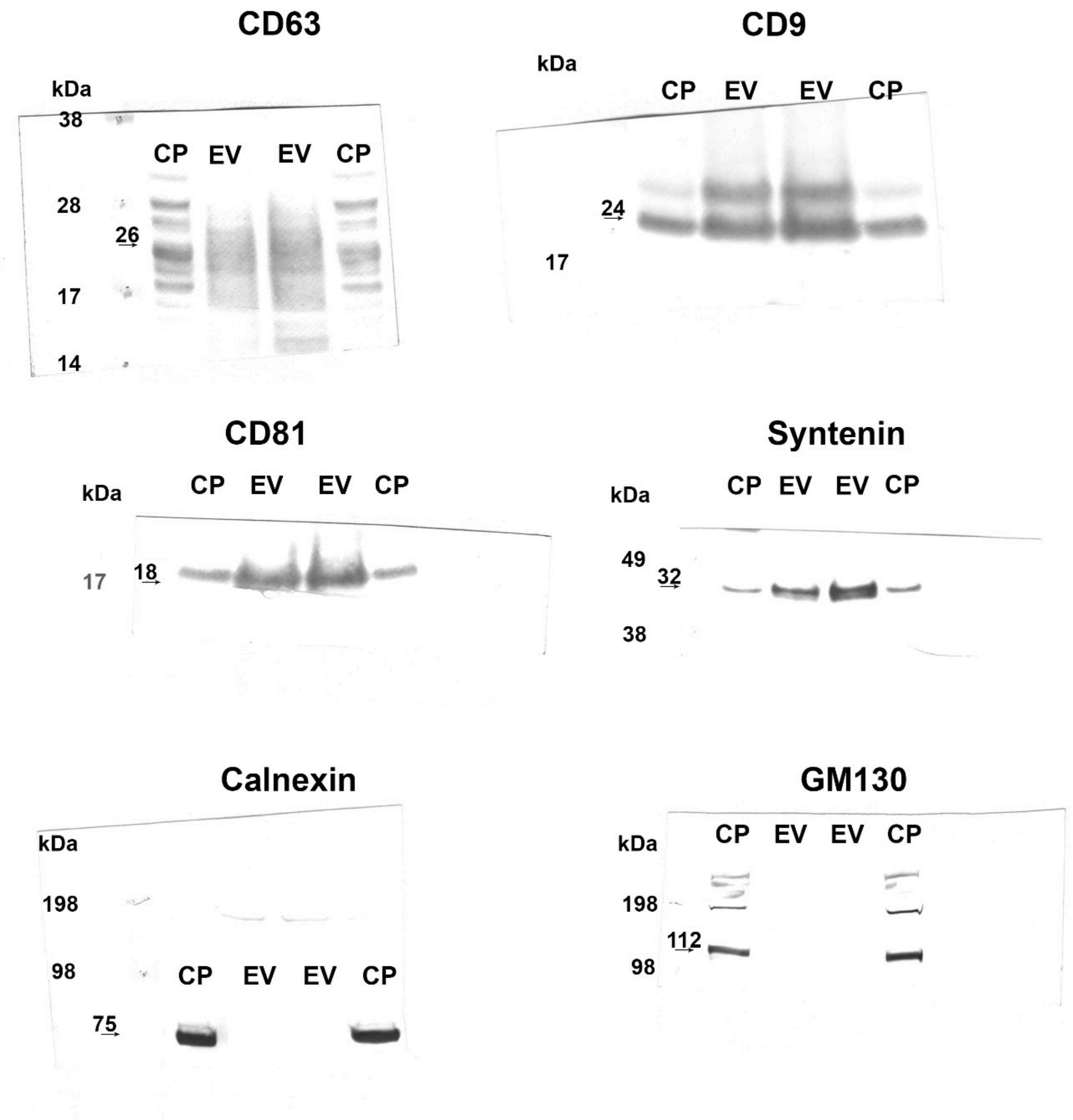

Figure S3. Full western blot membranes corresponding to Figure 1. Complete membranes displaying EV markers CD63, CD9, CD81, and syntenin, along with calnexin (an endoplasmic reticulum marker) and GM130 (a Golgi marker), in podocyte cell pellet (CP) samples and extracellular vesicles (EVs) derived from podocyte cultures. Molecular weight markers are indicated on the left (kDa), while arrows and the numbers shown above denote the molecular weight of the proteins of interest.

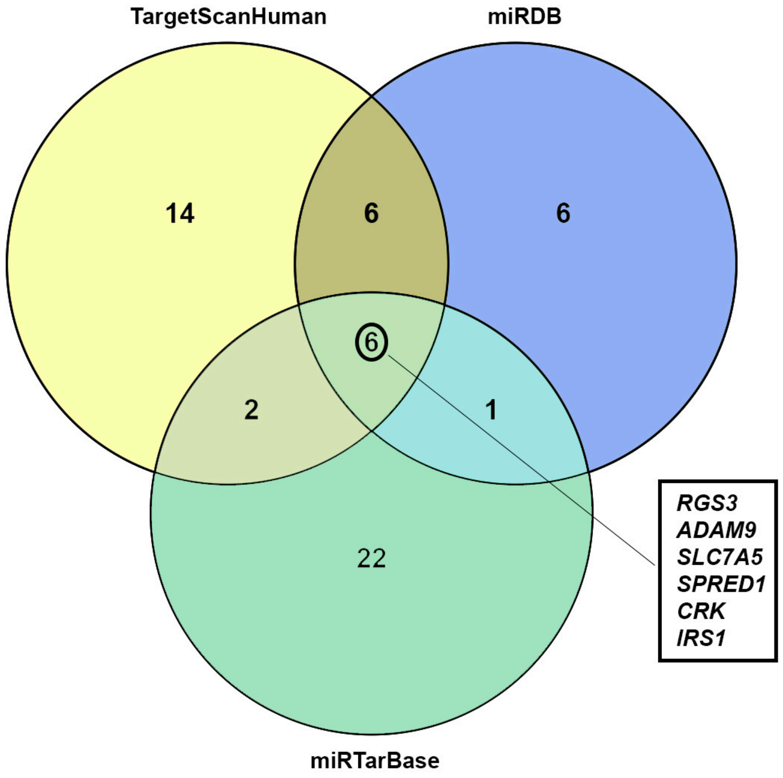

Figure S4. Venn diagram representing the common and unique predicted microRNA target genes across three distinct databases. The intersection of TargetScanHuman (yellow), miRDB (blue), and miRTarBase (green) databases revealed a core set of six common target genes (*RGS3, ADAM9, SLC7A5, SPRED1, CRK*, and *IRS1*). Non-common target genes include those uniquely identified by a single repository (14 for TargetScanHuman, 6 for miRDB, and 22 for miRTarBase) or shared exclusively between two databases (6 between TargetScanHuman and miRDB, 2 between TargetScanHuman and miRTarBase, and 1 between miRDB and miRTarBase). Gene symbols for the common targets are highlighted in the callout box.

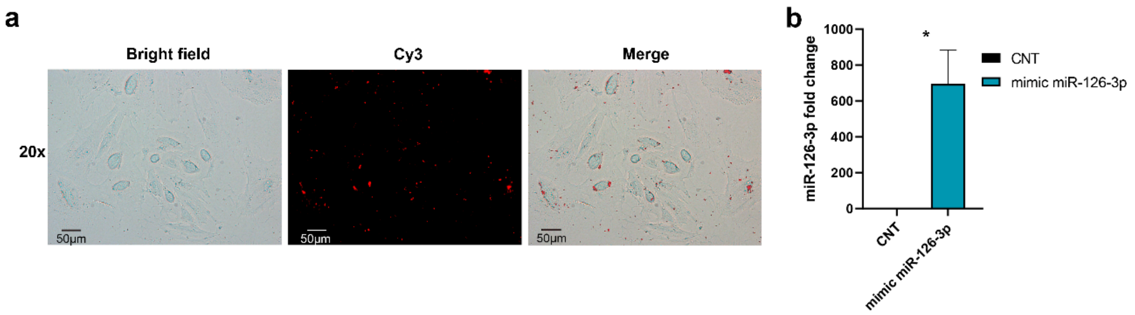

Figure S5. Assessment of miR-126-3p transfection efficiency in cultured podocytes. (a) Representative micrographs of podocytes transfected with Cy3-labeled control oligonucleotides. Bright field, Cy3 fluorescence, and merged images are shown, confirming cellular uptake of the transfected material. (b) RT-qPCR analysis revealed a significant elevation of intracellular miR-126-3p in cells transfected with the miR-126-3p mimic compared to the control group (CNT), validating the efficiency of transfection. Results are expressed as mean ± standard deviation (SD) of the fold change relative to controls. Statistical significance was assessed using an unpaired Student's t-test. $P < 0.05$ was considered statistically significant.. *$p < 0.05$.

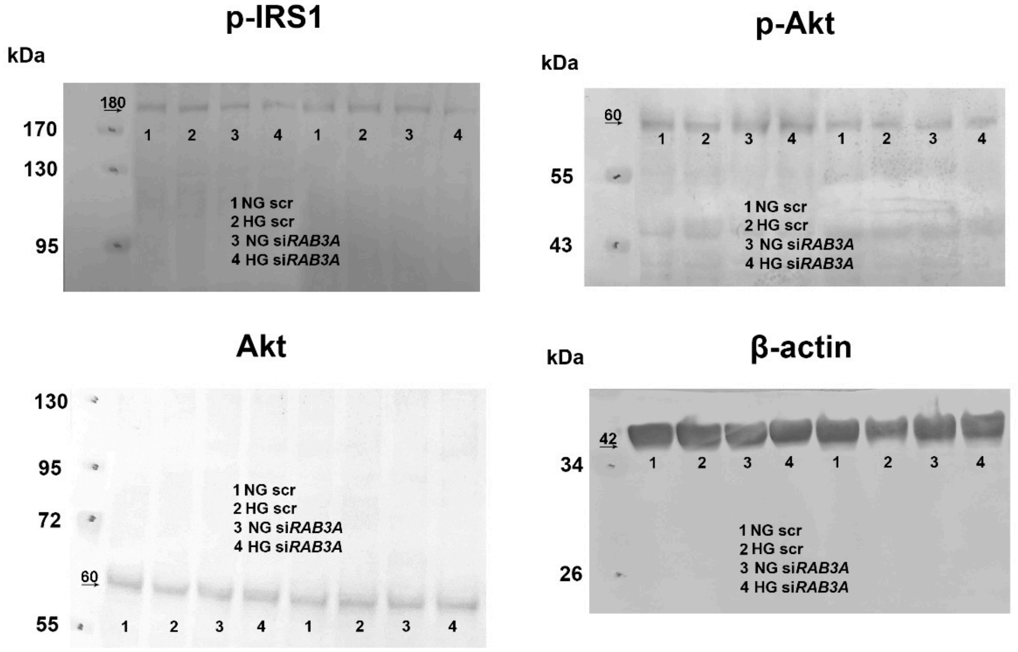

Figure S6. Full western blot membranes corresponding to Figure 4. Complete western blot membranes showing the expression of phosphorylated IRS1 (p-IRS1), phosphorylated Akt (p-Akt), and total Akt in treated and/or silenced podocyte samples. Molecular weight markers are indicated on the left (kDa). Arrows indicate the bands corresponding to the proteins of interest, and the numbers above the arrows indicate their expected molecular weights.
